# Anticoronavirus and Immunomodulatory Phenolic Compounds: Opportunities and Pharmacotherapeutic Perspectives

**DOI:** 10.3390/biom11081254

**Published:** 2021-08-23

**Authors:** Naiara Naiana Dejani, Hatem A. Elshabrawy, Carlos da Silva Maia Bezerra Filho, Damião Pergentino de Sousa

**Affiliations:** 1 Department of Physiology and Pathology, Federal University of Paraíba, João Pessoa 58051-900, Brazil; naiaradejani@gmail.com; 2 Department of Molecular and Cellular Biology, College of Osteopathic Medicine, Sam Houston State University, Conroe, TX 77304, USA; hxe007@shsu.edu; 3 Department of Pharmaceutical Sciences, Federal University of Paraíba, João Pessoa 58051-900, Brazil; carlosmaia1996@gmail.com; 4 Postgraduate Program in Bioactive Natural and Synthetic Products, Federal University of Paraíba, João Pessoa 58051-900, Brazil

**Keywords:** natural products, flavonoid, plants, chalcone, Middle East Respiratory Syndrome Virus, SARS-CoV, MERS-CoV, SARS-CoV-2, COVID-19, viruses

## Abstract

In 2019, COVID-19 emerged as a severe respiratory disease that is caused by the novel coronavirus, Severe Acute Respiratory Syndrome Coronavirus-2 (SARS-CoV-2). The disease has been associated with high mortality rate, especially in patients with comorbidities such as diabetes, cardiovascular and kidney diseases. This could be attributed to dysregulated immune responses and severe systemic inflammation in COVID-19 patients. The use of effective antiviral drugs against SARS-CoV-2 and modulation of the immune responses could be a potential therapeutic strategy for COVID-19. Studies have shown that natural phenolic compounds have several pharmacological properties, including anticoronavirus and immunomodulatory activities. Therefore, this review discusses the dual action of these natural products from the perspective of applicability at COVID-19.

## 1. Introduction

Coronaviruses (CoVs) are positive single-stranded (+ss) RNA viruses belonging to family Coronaviridae [1]. A large number of CoVs have been discovered as the causative agents of diseases in animals and humans [2]. Seven human CoVs (HCoVs) were discovered to date and they have all been linked to respiratory diseases. Four HCoVs cause mild diseases; whereas three HCoVs are the causative agents of severe respiratory diseases [3,4]. Of those three HCoVs, Severe Acute Respiratory Syndrome-CoV (SARS-CoV) was the first discovered in 2002–2003, followed by Middle East Respiratory Syndrome-CoV (MERS-CoV) in 2012, and finally the causative agent of the current COVID-19 pandemic; SARS-CoV-2 in 2019 [3,4]. SARS-CoV-2 was first discovered in patients that were linked to Huanan Seafood Market in Wuhan, China [4]. Since its emergence according to World Health Organization (WHO), millions of COVID-19 cases have been reported worldwide with over 4 million deaths. The severity of the diseases associated with SARS-CoV, MERS-CoV, and SARS-CoV-2 and the high fatality rates have prompted several research groups to develop effective antivirals against coronaviruses. Natural products have shown antiviral activities against several viruses including coronaviruses [5]. Of these natural products, phenolic compounds have shown a wide range of pharmacological activities [6].

Phenolic compounds are chemically characterized by having at least one aromatic rings attached to one or more hydroxyl substituent, and more than 8000 phenolic compounds have already been identified in plants [7]. Several plant families contain phenolic compounds including Sapindaceae [8], Rubiaceae [9], Crassulaceae [10], Punicaceae [11], Fabaceae [12], and others.

**Figure 1:**
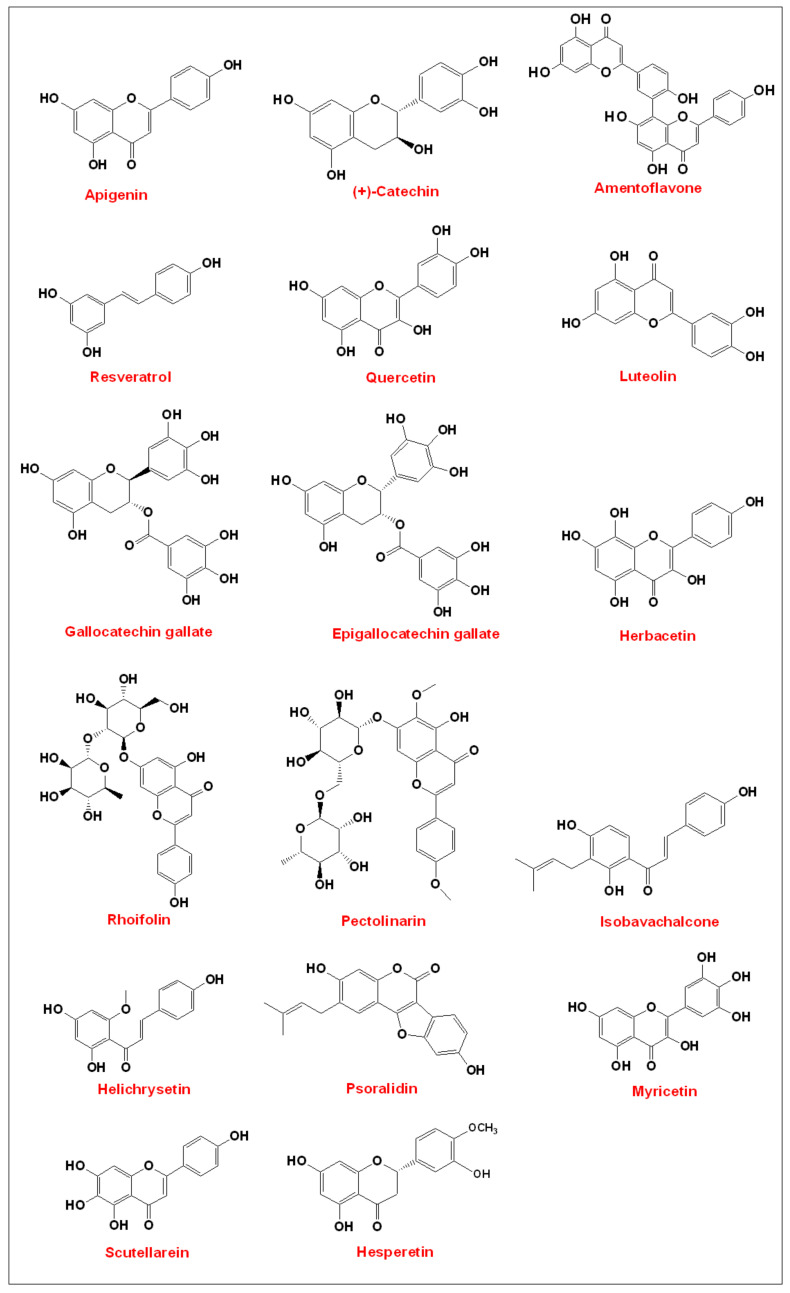
Chemical structures of anticoronavirus phenolic compounds found in nature.

Flavonoids are large group of secondary metabolites produced by a wide range of botanical families and are found in several plant parts. In fact, there are many flavonoids that are also phenolic compounds [13]. These natural products are synthesized by the phenylpropanoid pathway and are categorized into different classes based on structure, degree of hydroxylation, and polymerization [13]. Also, several activities have been reported including antimicrobial, antioxidant, anti-inflammatory, and antiviral activities [13]. Flavonoids have been tested for their antiviral activities since 1951 [14]. Quercetin, among other flavonoids, showed antiviral effects against influenza A virus, herpes simplex virus type 1, respiratory syncytial virus (RSV and other viruses [15,16,17,18,19]. The first study to document the anticoronavirus activity of flavonoids was published in 1990 [20]. In this study, quercetin (Figure 1) inhibited the replication of human coronavirus-OC43 (HCoV-OC43) and neonatal calf diarrhea coronavirus (NCDCV) in embryonic bovine lung fibroblasts. In another study, flavonoids inhibited the replication of porcine epidemic diarrhea virus (PEDV) [21]. Therefore, this review discusses the immunomodulatory activities of natural phenolic compounds, mainly flavonoids, that have antiviral activity against SARS-CoV, MERS-CoV, and SARS-CoV-2. These compounds could be further developed into more effective drugs for the treatment of COVID-19. Figure 1 illustrates these compounds.

## 2. Materials and Methods

The present study was carried out based on the literature review of natural phenolic compounds, immunomodulatory action and coronavirus. The search, performed in the PubMed database, concerning studies published until December 2020, used the following keywords: coronavirus, phenol, phenolic compounds, immunomodulatory, Middle East Respiratory Syndrome Virus, 229E, NL63, OC43, HKU1, SARS-CoV, MERS-CoV or SARS-CoV-2 (2019-nCoV or COVID-19). The scientific publications on immunomodulatory phenolic compounds found in nature and against coronaviruses were selected from studies published in English and discussed in this manuscript.

## 3. Flavonoids as Entry Inhibitors for SARS-CoV

Since the emergence of SARS-CoV in 2002–2003, several groups have been testing plant-derived compounds for anti-SARS-CoV activity. Screening extracts from 121 chinese herbs, for binding to SARS-CoV S2 domain and inhibiting viral infection of target cells, identified luteolin as a flavonoid with anti-SARS-CoV activity [22]. Luteolin inhibited SARS-CoV entry into Vero E6 cells with a concentration that results in 50% inhibition (IC_50_) of 10.6 μM and concentration that reduces cell viability by 50% (CC_50_) of 155 μM. Luteolin exerts its anti-SARS-CoV activity by binding to S2 domain of SARS-CoV S protein and inhibiting viral envelope fusion with cellular membranes [22]. In the same study, quercetin inhibited HIV-luc/SARS pseudotyped virus entry into Vero E6 cells with an IC_50_ of 83.4 μM and a high CC_50_ of 3320 μM indicating that different flavonoids could be used as effective and safe inhibitors of SARS-CoV viral infections.

Another study showed that the *n*-butanol fraction from the dried bark of *Cinnamomum cassia* which contain flavonoids inhibited both HIV/SARS-CoV pseudovirus infection and wild-type SARS-CoV infection of target cells [23].

## 4. Flavonoids as SARS-CoV and MERS-CoV Protease Inhibitors

The coronaviruses’ genome codes for two proteinases, 3-chymotrypsin-like protease (3CL^pro^) and papain-like protease 2 (PL^pro^), that are critical for viral polyprotein processing that precedes viral replication [24]. These critical roles of 3CL^pro^ and PL^pro^ in viral life cycle has prompted many research groups to screen for and developing antiviral drugs that inhibit these two proteinases. Flavonoids such as hesperetin, quercetin, and naringenin were tested for their inhibitory effects against SARS-CoV 3CL^pro^ in cell-free and cell-based assays. However, only hesperetin inhibited 3CL^pro^ with an IC_50_ of 60 μM and 8.3 μM in cell-free and cell-based assays, respectively [25]. The poor water solubility of hesperetin may explain its lower efficacy in cell-free assays. Quercetin was used as a control compound to evaluate the inhibitory effect of compounds, isolated from the medicinal plant *Torreya nucifera*, on commercial 3CL^pro^ [26]. In this study, the biflavone amentoflavone showed the highest inhibitory activity with an IC_50_ of 8.3 μM. However, quercetin, luteolin, and apigenin showed lower inhibitory activity on 3CL^pro^, compared to amentoflavone, with IC_50_ of 23.8, 20.2, and 280.8 μM respectively. Other flavonoids, such as gallocatechin gallate (GCG) and epigallocatechin gallate (EGCG) inhibited recombinant SARS-CoV 3CL^pro^ with the galloyl moiety at 3-OH position reported as being important for the inhibitory activity [27]. The IC_50s_ were 47 and 73 μM for GCG and EGCG, respectively.

A flavonoid library was also tested to identify inhibitors for SARS-CoV 3CL^pro^. In this study, herbacetin, rhoifolin and pectolinarin inhibited recombinant SARS-CoV 3CL^pro^ and the IC_50_ were reported as 33.17, 27.45 and 37.78 μM respectively [28].

With respect to PL^pro^, six flavonoids isolated from *Psoralea corylifolia* L. namely, bavachinin, neobavaisoflavone, isobavachalcone, 4′-*O*-methylbavachalcone, psoralidin and corylifol A inhibited SARS-CoV PL^pro^ in a fluorescence assay using the fluorogenic substrate, Z-RLRGG-7-amido-4-methylcoumarin [29]. Psoralidin and isobavachalcone were identified as the most active with IC_50_ of 4.2 ± 1.0 and 7.3 ± 0.8 μM respectively.

A study screened flavonoids for inhibition of MERS-CoV 3CL^pro^ and identified herbacetin, isobavachalcone, and helichrysetin as potent inhibitors with IC_50_ of 40.59, 35.85, and 67.04 μM respectively [30].

## 5. Flavonoids as Inhibitors of SARS-CoV NSP13 (Helicase/ATPase)

SARS-CoV nonstructural protein 13 (NSP13) possesses helicase and ATPase activity both of which are important for viral life cycle [31]. In addition to flavonoids activity against SARS-CoV 3CL^pro^, quercetin was identified as an inhibitor of NSP13 helicase activity with an IC_50_ of 8.1 μM [32]. Unlike other flavonoids, myricetin and scutellarein showed inhibition to ATPase activity of NSP13 with an IC_50_ of 2.71 ± 0.19 μM and 0.86 ± 0.48 μM, respectively [33]. The inhibition was specific to SARS-CoV ATPase of NSP13 as the compounds did not inhibit hepatitis C virus helicase.

## 6. Flavonoids and Other Natural Phenolic Compounds as Inhibitors of SARS-CoV-2

Since the emergence of SARS-CoV-2 in December 2019, several studies have focused on repurposing drugs that have been used for other health conditions including drugs with reported anti-SARS-CoV activity. In line with that, flavonoids that inhibited SARS-CoV were tested against SARS-CoV-2. In one study, quercetin and EGCG were shown to interact with and inhibit SARS-CoV-2 3CL^pro^ activity in a FRET-based enzymatic assay [34,35]. Moreover, EGCG inhibited the entry of SARS-CoV-2-pseudotyped virus and live SARS-CoV-2 into HEK293T-hACE2 and Vero cells respectively [36]. Another study has demonstrated that EGCG inhibited the endoribonuclease enzymatic activity of SARS-CoV-2 nonstructural protein-15 (Nsp15) with an IC_50_ of 1.62 μM, while blocking viral replication in Vero cells with an IC_50_, 0.2 μM [37]. The low IC_50_ of ECGC indicates its potency and warrants its further development as a potential SARS-CoV-2 antiviral. GCG was also found to inhibit the binding of SARS-CoV-2 N protein to viral RNA inhibiting viral replication in A549-hACE2 with an IC_50_, 44.4 μM [38]. As shown previously with SARS-CoV 3CL^pro^, herbacetin and pectolinarin inhibited SARS-CoV-2 3CL^pro^ with an IC_50_ of 53.90 and 51.64 μM, respectively [28,39]. However, rhoifolin exhibited weaker inhibition, whereas baicalin showed stronger inhibition of SARS-CoV-2 3CL^pro^ than that observed for SARS-CoV 3CL^pro^. These differences in inhibition of 3CL^pro^ by rhoifolin and baicalin may be attributed to the slight differences in the amino acid sequence since the two 3CL^pro^ have 96% sequence identity. A study has also demonstrated potent inhibition of recombinant SARS-CoV-2 3CL^pro^ by myricetin, which suggests that myricetin could be further tested and developed as a potential SARS-CoV-2 antiviral [40].

A recent study that screened for inhibitors of angiotensin converting enzyme 2 (ACE2), the SARS-CoV-2 receptor, identified the flavonoids rutin, quercetin, and tamarixetin as inhibitors of ACE2 activity [41]. However, the most potent of all flavonoids tested was quercetin with an IC_50_ of 4.48 μM.

Stilbene derivatives, such as resveratrol, are natural polyphenolic compounds that are abundant in a variety of plants including grapes [42]. They have a wide range of activities including antimicrobial, antioxidant, antileukemic, anti-platelet aggregative, protein tyrosine kinase inhibitory, anti-inflammatory, anticarcinogenic activity, antiviral activities [42]. Several studies evaluated resveratrol and its derivatives for their antiviral activity against SARS-CoV, SARS-CoV-2, and MERS-CoV [42,43,44]. Resveratrol and a few derivatives showed potent inhibition of SARS-CoV replication [42]. Resveratrol also inhibited MERS-CoV viral replication, nucleocapsid protein expression, and protected MERS-CoV infected cells from apoptosis [43]. Most recently, a study showed that resveratrol inhibited SARS-CoV-2 infection of Vero cells [44]. This study suggests that resveratrol inhibits the entry of virus into Vero cells. In addition to the above studies, several molecular docking and computational studies have described different flavonoids that target SARS-CoV-2 S protein, 3CL^pro^, PL^pro^, helicase and RNA polymerase [45,46,47,48,49,50,51,52,53,54,55,56,57,58,59,60,61,62]. Molecular docking studies have also identified resveratrol, quercetin, and luteolin as phenolic compounds that binds with high affinity to ACE2 receptor [63,64].

All the previous activities of flavonoids and phenolic compounds indicate that they may serve as promising and potential therapeutics for SARS-CoV-2 and could be considered for further development. Table 1 summarizes the anticoronavirus activities of phenolic compounds discussed in this study.

## 7. Immune Response to SARS-CoV-2

Immune responses to viral infections are essential to control viral replication, kill infected cells and induce protective immunity against virus [65,66]. Following infection, viral nucleic acid and viral proteins are detected by patter recognition receptors (PRRs), such as Toll-like receptors (TLRs) on immune cells and other cells [65]. The recognition of viral proteins and nucleic acid results in production of inflammatory cytokines, chemokines and adhesion molecules by immune tissue resident cells, such as macrophages [67]. Although appropriate levels of proinflammatory cytokines are required to activate immune cells involved in viral control, extremely high levels of IL-1β, IL-10, G-CSF, GM-CSF, IFN-γ and TNF-α were detected in COVID-19 patients [68]. Moreover, disease severity positively correlated with increased IL-6 levels [69].

Type I interferon (IFN) is required to activate cellular antiviral mechanisms to suppress viral replication and virion assembly [70]. Severe COVID-19 patients demonstrated decreased type I IFN response and exacerbated inflammation [71]. Indeed, autoantibodies against type I IFN were detected in severe COVID-19 pneumoniae [72] and mutations in genes related to type I IFN immunity were also detected in critical patients [73]. Besides the host defects in type I IFN immunity, a study showed that SARS-CoV-2 ORF6 protein inhibited type I interferon production and signaling pathway [74]. In addition, natural killer (NK) cell numbers were reduced in blood of severe COVID-19 patients [75]. Therefore, impaired innate immune responses and increased production of proinflammatory cytokines may contribute to disease severity and worse outcomes in COVID-19 patients.

In addition to innate immune system deficiencies reported in severe COVID-19 patients, adaptive immune responses are also compromised as demonstrated by lymphopenia and decreased numbers of T cells in severe COVID-19 patients [76]. Direct viral cytotoxicity, impaired cell proliferation and enhanced apoptosis have been related to lymphopenia in severe COVID-19 patients [77]. Increased levels of C-reactive protein (CRP), D-dimer, fibrinogen, procalcitonin, lactate dehydrogenase (LDH), and ferritin have also been reported in severe COVID-19 patients [78,79]. The immune dysregulation during COVID-19 results in hyperinflammation, pulmonary injury, coagulopathy and multiorgan dysfunction, leading to worse outcome [80]. Indeed, comorbidities, including hypertension, diabetes and obesity, are prevalent in severe COVID-19 patients [81,82,83].

Natural products with immunomodulatory activities are worth investigation as promising therapeutics for COVID-19. In addition to antiviral activity, many natural products have antifungal and antibacterial activities, which are interesting since coinfections have been reported in severe SARS-CoV-2 pneumonia patients [84]. Anti-inflammatory, antiapoptotic, antioxidant, and immunomodulatory activities have been described for natural compounds [85,86,87,88]. Indeed, natural compounds capable of reducing inflammation without compromising host immunity would be beneficial for treatment of severe COVID-19 [80]. Herein, we review the immunomodulatory activities of natural phenolic compounds, including flavonoids, that possess anti-SARS-CoV, anti-MERS-CoV, and anti-SARS-CoV-2 activities.

## 8. Immunostimulatory Activities of Natural Phenolic Compounds

A variety of natural compounds have shown anti-inflammatory and antioxidant activities in addition to immunomodulatory activities that are reported in different experimental models [87,88,89] (Table 2). Apigenin and luteolin at 10 μM induced activation of NK and CD8^+^ T cells (CTLs) in vitro, and enhanced the proliferation of splenocytes stimulated with lipopolysaccharide (LPS) [90].

EGCG enhanced the antiviral state in Huh7 cells, a hepatoma cell line, infected with hepatitis C virus (HCV) [91]. Treatment of HCV-infected Huh7 cells with 10 μM of EGCG enhanced polyinosinic–polycytidylic acid (Poly I:C) induced expression of IFN-stimulated genes (ISGs), increased TLR3 and IFN-λ1 expression, and decreased viral replication [91]. Indeed, pretreatment of Huh7 cells with 10 μM epigallocatechin gallate followed by HCV dsRNAs enhanced antiviral defense that is mediated by interferon-λ1 (IFN-λ1), TLR3, RNA-sensing retinoic acid-inducible gene I (RIG-I) and IFN-stimulated gene (ISG) expression [92]. In a murine leukemia model, oral treatment with 87.26 μmol/kg of EGCG induced T and B cell proliferation and NK cell activity [93]. Furthermore, EGCG (50 μM) increased macrophage receptor with collagenous structure (MARCO) expression and improved macrophage phagocytosis of *Streptococcus pneumoniae* [94]. Ex vivo experiments using cells from mice orally treated, every day for 6 weeks, with 1000 mg/kg of EGCG fraction of green tea extract demonstrated enhanced innate and adaptive immune responses such as NK cytolysis, peritoneal cells phagocytosis, splenocyte proliferation, and IL-2 and IFN-γ production [95].

Quercetin, resveratrol and apigenin were also reported to have antimicrobial and immunostimulatory activities [88,96,97]. Mice fed with quercetin (0.86 μmol day^−1^) for 34 days and immunized at day 29 with forssman heterophilic glycolipid antigen, a T cell- dependent antigen, showed increased ex vivo B and T cell proliferation as well as enhanced numbers of IgM-producing lymphocytes [98]. During in vitro viral infection, macrophages treated with a noncytotoxic concentration of quercetin (100 μM) showed impaired dengue virus type 1 and type 3 (DENV1, DENV3) replication and diminished TNF-α and IL-6 secretion by human U937-DC-SIGN macrophages in the presence or absence of enhancing 4G2 antibodies, whereas resveratrol (100 μM) and apigenin (40 μM) only impaired DENV3 replication in the absence of enhancing antibodies [96]. On the other hand, quercetin (10 μM) and resveratrol (50 μM) suppressed human metapneumovirus (hMPV) replication, decreased 8-isoprostane, an oxidative stress marker, and reduced IL-8, RANTES, IL-6, TNF-α, CXCL-10, CCL4 production by hMPV-infected A549 airway epithelial cell line [99].

Resveratrol at 25 μM inhibited influenza virus replication through activation of TLR9/MyD88/IRF7 pathway in A549 infected cells, and enhancing IFN-β production [97]. Also, pre-treatment of RAW 264.7 cells with 100 μg/mL of aqueous extract of *Eupatorium fortune* demonstrated antiviral activity against influenza A virus infection by enhancing production of type I IFN. Indeed, quercetin was identified as one of the active antiviral and immunomodulatory compounds of the extract [100]. This was confirmed by a study which showed that pre-treatment with 3.0 μg/mL quercetin inhibited influenza virus replication in RAW 264.7 cells and increased IFN-β production [101]. Moreover, quercetin dose dependently decreased nontypeable *Haemophilus influenzae* (NTHi) bacterial viability in vitro, reduced production of proinflammatory markers in the lungs of infected mice that were pre-treated with 60 mg/kg for 8 days and for 24h postinfection, and decreased mortality of NTHi-infected zebrafish that were intraperitoneally treated with 0.3 mg/g of quercetin at 29 and 53h postinfection [102].

Hesperetin is another flavonoid with antioxidant, anti-inflammatory, anticancer and antimicrobial activity [103,104]. It has been shown that hesperetin (25 μM) activated host cellular and humoral responses [105], enhanced LPS-mediated in vitro proliferation of splenocytes, and potentiated killing activity of NK and CTLs [105]. Moreover, hesperetin activated antigen presenting cells (APCs), enhanced CTL response, and antitumor immunity when used as an adjuvant at 2.65 μmol/mouse in combination with inactivated B16F10 melanoma cells vaccine which prolonged the survival of tumor-bearing mice [106].

**Table 2:** Immunostimulatory effects of natural phenolic compounds.

Compound	Experimental Model		Dose/Concentration	Effect	Reference
Apigenin Luteolin	In vitro	LPS-stimulation of murine splenocytes Killing of target tumor cells	10 μM 1–10 μM	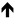 Proliferation of splenocytes 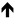 CTL and NK cytotoxicity activity	[90]
Epigallocatechin gallate (EGCG)	In vitro	HCV JFH-1-infected Huh7 treated with EGCG 1h prior to poly I:C stimulation	1–10 μM	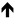 Poly I:C induced expression of ISGs 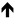 TLR3 and IFN-λ1 expression 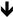 Virus replication	[91]
	In vivo	Murine leukemia model	10.91, 43.63 and 87.26 μmol/kg	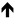 T and B cell proliferation 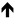 NK activity 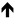 Macrophage phagocytosis	[93]
	In vitro	Human U937-DC-SIGN macrophages infected with DENV1 or DENV2	100 μM	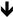 DENV1 and DENV2 replication 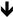 TNF-α and IL-6 secretion	[96]
	Ex vivo	LPS-stimulated proliferation of B cells SRBC-immunized mice	215.1 μmol/kg in diet for 34 days	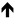 B cells proliferation 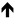 IgM-producing lymphocytes	[98]
Quercetin Resveratrol	In vitro	hMPV-infected A549 airway epithelial cell line	10 μM 50 μM	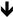 Oxidative stress 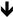 IL-8, RANTES, IL-6, TNF-α, CXCL-10, CCL4 secretion 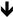 Virus replication	[99]
Hesperetin	In vivo	Adjuvant in combination with inactivated B16F10 melanoma cells vaccine	2.65 μmol/mouse	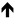 APC activation 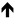 CTL response	[106]

LPS: Lipopolysaccharide. CTL: Cytotoxic T Lymphocytes. NK: Natural Killer Cells. SRBC: Forssman heterophilic glycolipid antigen occurring on sheep erythrocytes. DENV: Dengue virus. hMPV: Human metapneumovirus. APC: Antigen Presenting Cells. The compounds in the table are in the order in which the compounds are presented in the section *Immunostimulatory Activities of Natural Phenolic Compounds*. 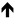 Increased or 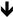 decreased.

## 9. Effects of Natural Phenolic Compounds on NF-κB Pathway and Inflammation

It has been established that activated nuclear factor-κB (NF-κB) translocates to the nucleus and induces the transcription of genes involved in inflammation, apoptosis, cell proliferation, survival, and differentiation [107]. Since NF-κB drives the expression of cytokines and others inflammatory mediators involved in COVID-19 hyperinflammatory state, targeting NF-κB pathway has been proposed to ameliorate severe inflammation in COVID-19 [108,109].

Monocyte-derived macrophages are involved in lung and multiorgan inflammation observed in severe COVID-19 patients [110,111,112] which necessitate the investigation of potential natural phenolic compounds that could reduce NF-κB activation and inhibit the production of proinflammatory cytokines and chemokines by macrophages.

The anti-inflammatory activity of some natural compounds is due to their ability to impair NF-κB activation. The in vitro suppression of NF-κB pathway by amentoflavone, herbacetin, rhoifolin, luteolin, myricetin, psoralidin, scutellarin and hesperetin has been described in RAW 264.7 murine macrophages. Amentoflavone decreased NO production by LPS-activated RAW 264.7 macrophages, and this activity was dose dependent [113,114]. RAW 264.7 cells, that were pretreated with 60 μM of amentoflavone, showed reduced NF-κB activation and translocation of p65 to the nucleus. Moreover, inducible nitric oxide synthase (iNOS) expression and NO production were reduced in these cells [113]. Herbacetin exerts its anti-inflammatory effects by inhibiting Jun N-terminal kinase (JNK) and NF-κB signaling pathway in RAW 264.7. Herbacetin (50 μM) reduced the production of NO, IL-1β and TNF-α in cells that are stimulated with LPS [115]. Rhoifolin (100 μmol/L) suppressed IκBα and IKKβ phosphorylation in RAW 264.7 cells, that are stimulated with LPS, resulting in reduced production of TNF-α, IL-1β and IL-6 cytokines, and lower levels of iNOS and CCL2 mRNA [116]. Furthermore, luteolin (5 μM) impaired NFκB translocation in LPS-activated RAW 264.7 cells, induced heme oxygenase-1 (HO-1) expression, and reduced iNOS expression and NO production [117]. Similarly, myricetin (100 μM) impaired STAT-1 activation, IκBα degradation, and the p65 nuclear translocation, and induced heme HO-1 expression in LPS-stimulated RAW 264.7 cells [118]. Psoralidin (30 μM) inhibited iNOS expression in LPS-activated RAW 264.7 cells by suppressing IKK phosphorylation, IκB degradation and NF-κB nuclear translocation [119]. In addition, pretreatment with 100 μM scutellarin decreased the production of prostaglandin E_2_ (PGE_2_), NO, IL-6 and TNF-α by LPS-activated RAW 264.7 cells [120]. Hesperetin exerts anti-inflammatory effects in LPS-stimulated RAW 264.7 since treatment with 40 μM decreased TNF-α, IL-6, IL-1β production, and reduced iNOS and COX-2 expression by impairing NF-κB activation and stimulation of HO-1 and nuclear factor erythroid 2-related factor 2 (Nrf2) pathways [121].

In human monocytes, 10 μM of apigenin decreased IL-1β, TNF-α and IL-8 production by cells stimulated with LPS. This anti-inflammatory activity of apigenin is due to inhibition of NF-κB activation by reducing the phosphorylation of p65 and inhibiting IKK [122]. Another study have shown that pre-treatment of LPS-stimulated THP-1-derived macrophages, with 25 μM of apigenin, blocked ERK1/2 phosphorylation, impaired NF-κB activation, and decreased the expression of chemokine (C-C motif) ligand 5 (CCL5), intercellular adhesion molecule-1 (ICAM-1), vascular cell adhesion protein-1 (VCAM-1), IL-1β, and IL-6 [123].

Also, THP-1 macrophages that were pre-treated with 40 μM catechin, before infection with *Porphyromonas gingivalis,* showed downregulation of NF-κB activation, and reduced IL-1β and TNF-α production with no effect on bacterial growth [124].

EGCG can directly bind to CXCL9, 10 and 11 chemokines and limit their ability to recruit leukocytes [125]. In addition, pre-treatment with 10 μM EGCG, quercetin and luteolin reduced inflammation in endothelial cells by impairing IKKB activation and downregulating VCAM-1 expression [126]. Another study have demonstrated that EGCG and GCG in a concentration dependent manner (0.3 and 30 μM) blocked IκBα degradation, NF-κB activation and IL-12p40 production in LPS-stimulated murine peritoneal macrophages and J774.1 macrophages, and these effects were dose dependent [127]. Similarly, luteolin (10 and 100 μM) inhibited VCAM-1 expression on formyl-MLP (fMLP)-stimulated endothelial cells [128]. In the same study, luteolin suppressed the adhesion of monocytes to endothelial cells by reducing chemokine monocyte chemotactic protein-1 (MCP-1), ICAM-1 and VCAM-1 expression by endothelial cells stimulated with TNF-α. Such effects were explained by the ability of luteolin to inhibit NF-κB activation by impairing IκBα degradation, IκB kinase β (IKKβ) expression, and NF-κB nuclear translocation in endothelial cells [129]. Another study showed that isobavachalcone impaired NF-κB activation and ICAM-1 expression in a cerebrovascular endothelial cell line that was stimulated with LPS, polyriboinosinic polyribocytidylic acid (Poly [I:C]) or macrophage-activating lipopeptide 2-kDa (MALP-2). In line with the above findings, as well as at 0,1, 1 or 5 μM isobavachalcone inhibited the adhesion of monocytes to LPS-stimulated endothelial cells in vitro [130].

Studies showed that resveratrol impaired NF-κB activation in different cells including myeloid cells, HeLa, and Jurkat cells that were stimulated with phorbol myristate acetate (PMA), LPS, H_2_O_2_, okadaic acid or ceramides [131]. Indeed, human and murine macrophages stimulated with TNF-α or LPS in the presence of 25 μM of resveratrol showed reduced production of proinflammatory cytokine and chemokine [132]. In human epithelial cells, high concentration of resveratrol (300 μM) inhibited rhinovirus replication and ICAM-1 expression, and decreased basal levels of IL-6 and RANTES in uninfected human epithelia [133].

Impaired NF-κB and reduced production of AP-1-dependent proinflammatory cytokines were described in LPS-stimulated RAW 264.7 macrophages that were pre-treated with 20 μM of quercetin. The ability of quercetin to impair TLR4/MyD88/PI3K downstream signaling pathways resulted in reduced production of NO, PGE_2_, TNF-α, IL-6, IL-1β and GM-CSF [134]. Interestingly, synergistic anti-inflammatory activity of quercetin and catechin was detected in LPS-stimulated RAW 264.7 macrophages that were treated with 3 μM of quercetin and 75 μM of catechin [135]. Pre-treatment with quercetin inhibited nuclear translocation of NF-κB p65 in human peripheral blood mononuclear cells (PBMCs) that were stimulated with oxidized low-density lipoprotein (OxLDL). Moreover, quercetin (25 μM) decreased PGE_2_ and IL-6 production, and downregulated TLR2 and TLR4 expression in these PBMCs [136]. The anti-inflammatory effects of hesperetin and resveratrol (100 μM) were reported in PBMCs that were stimulated with LPS. Pre-treatment with hesperetin or resveratrol, 2 h prior to stimulation, reduced the production of TNF-α, IFN-γ, CCL-2, CCL-5, IL-1β and GM-CSF, while only resveratrol inhibited IL-6 production [137].

BV-2 cells, a mouse microglial cell line, were used to study neuroinflammation in vitro [138]. Isobavachalcone, at 5 μM, suppressed p65 translocation to the nucleus and NF-κB activation in LPS-stimulated BV-2 cells, resulting in decreased expression of TNF-α, IL-6, IL-1β, and iNOS [139]. Scutellarin showed similar effects in LPS-stimulated BV-2 cells. In this study, 139.7 μM of scutellarin reduced AKT, JNK, p38 and p65 phosphorylation and suppressed the production of NO, TNF-α, IL-1β, and IL-6 [140].

Studies showed that helichrysetin possessed anti-inflammatory, anti-oxidant and anti-tumor activities in different cell lines [141,142]. Helichrysetin (50 μM) impaired NF-κB activation in mouse pancreatic β-MIN-6 cells [141], HeLa, and T98G cells [142]. Rheumatoid arthritis fibroblast-like synoviocytes treated for 48h with 10 or 20 μM of pectolinarin showed decreased activation of the phosphatidylinositol 3 kinase/protein kinase B pathway, reduced cell proliferation and decreased production of IL-6, IL-18, NO and PGE_2_ [143]. However, in LPS-stimulated RAW 264.7 macrophages, pectolinarin at 1, 10, 25 or 50 μM did not affect COX-2 expression and PGE_2_ synthesis [144]. Table 3 summarizes the anti-inflammatory activities of phenolic compounds discussed in this study.

## 10. Inhibitory Effects of Natural Phenolic Compounds on NLRP3 Inflammasome

Viral nucleic acids are recognized by PRRs, such as TLR 3,7,8 in the endosomes [145]. Recognition of viral proteins and nucleic acid by PRRs triggers myeloid differentiation primary response 88 (MyD88) and TIR-domain-containing adapter-inducing interferon-β (TRIF) signaling pathways culminating in the activation of interferon-regulatory factor 3/7 (IRF) and NF-κB transcription factors resulting in expression of pro-IL1β and pro-IL-18 [107]. Moreover, activation of cytosolic NOD-like receptor (NLR) family pyrin domain-containing 3 (NLRP3) inflammasome by pathogens, including viruses, results in activation of caspase-1, and consequently the processing of pro- IL-1β and pro-IL-18 into mature IL-1β and IL-18 [146,147]. It is noteworthy to mention that the activation of inflammatory caspases can induce a type of cell death called pyroptosis, which may be involved in exacerbated production of inflammatory cytokines during acute phase of COVID-19 [68,148]. Those events are important in the defense against infectious diseases but could promote inflammation, death and tissue injury.

**Figure 2:**
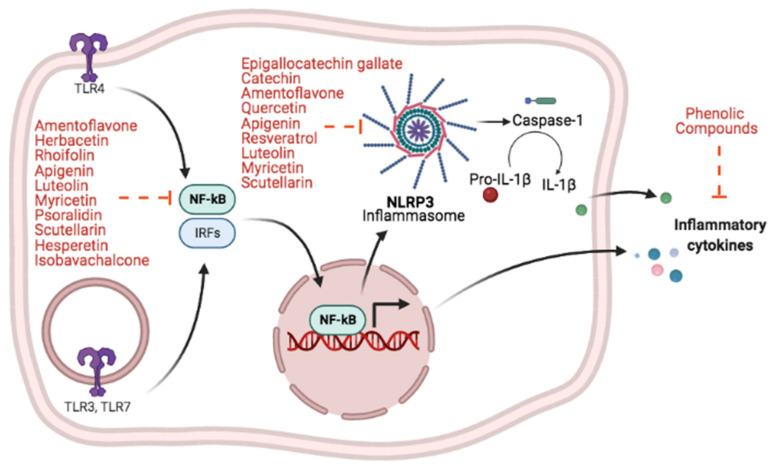
Natural phenolic compounds target NF-κB and NLRP3 pathways. Some compounds inhibit the activation or translocation of NF-κB to the nucleus, as well as inhibit NLRP3 inflammasome assembly. These actions impair the expression of inflammatory cytokines and the secretion of mature IL-1β. The inhibitory activities of natural phenolic compounds on NLRP3 inflammasome and NF-κB signaling pathways ameliorate exacerbated immune activation and reduces proinflammatory cytokines production during infections (Dashed lines = Inhibition. Green, blue, pink and gray balls represent inflammatory cytokines = IL-1β, IL-6, TNF-α and IL-12, respectively).

LDH is a marker for pyroptosis and is induced in severe COVID-19 patients [79]. Moderate and severe COVID-19 patients showed enhanced NLRP3 activation in PBMCs and lungs, which positively correlated with the severity of disease [149]. Treatments targeting NLRP3 inflammasome have been suggested to mitigate COVID-19-associated inflammation and complications [150]. Natural inhibitors of NLRP3 activation have been described, for example, amentoflavone [151], quercetin [152], apigenin [123], catechin [124], resveratrol [153], luteolin [154], scutellarin [155], epigallocatechin gallate [126], and myricetin [156] (Figure 2).

A study has shown that quercetin, at 100 μM, inhibited caspase-recruitment domain (ASC) oligomerization and NLRP3 inflammasome activation resulting in decreased IL-1β production by in vitro-stimulated macrophages [152]. Moreover, in a Kawasaki disease experimental model, treatment of mice with 100 mg/kg of quercetin prevented vascular inflammation and IL-1β production [152]. It was also found that treatment of macrophages with 25 μM apigenin blocked caspase-1 activation by targeting ASC and impairing NLRP3 inflammasome assembly [123]. Endoplasmic reticulum (ER) stress induced by palmitate in EA.hy926 cells, a hybridoma line derived from human endothelium and A549/8 cells, led to NLRP3 activation, IL-1β production and endothelial cell dysfunction. However, treatment of EA.hy-926 cells with 10 μM of quercetin, luteolin or epigallocatechin gallate reduced reactive oxygen species (ROS) production and thioredoxin-interacting protein (TXNIP) and NLRP3 inflammasome activation, resulting in lower IL-1β expression [126]. Moreover, EGCG (25 μM) reduced nucleus pulposus cell inflammation and cell death, induced by H_2_O_2,_ by interfering with cGAS/Sting/NLRP3 pathway [157].

Resveratrol, at 5 μM, inhibited assembly and activation of NLRP3 inflammasome in stimulated macrophages [153]. Also, resveratrol (30 μM) inhibited NLRP3 and IL-1β expression in BV-2 cells, and protected septic mice from encephalopathy by targeting NLPR3 at a concentration of 30 mg/kg [158].

Amentoflavone (10 µM) inhibited NLRP3 inflammasome activation in LPS-stimulated BV-2 cells [151]. Luteolin at a low concentration (2 μM) impaired NLRP3, ASC and caspase-1 expression by LPS-stimulated RAW 264.7 macrophages, and polarized macrophages into M2 macrophages by enhancing the expression of Arg-1 and IL-10, and decreasing M1 markers expression, including TNF-α, IL-6 and iNOS [154]. In addition, myricetin (75 µM) inhibited NLRP3 activation by blocking ASC oligomerization in macrophages [156].

Scutellarin has also been shown to inhibit NLRP3 inflammasome activation in different experimental models [155,159,160,161,162,163]. Treatment of LPS-primed bone-marrow derived macrophages (BMDMs) with 400 μM of scutellarin followed by ATP resulted in enhanced PKA signaling, reduction of ASC oligomerization, impaired caspase-1 activation, and lower IL-1β production compared to BMDMs that were not treated with scutellarin [155]. Figure 2 illustrates the anti-inflammatory activities of natural phenolic compounds by targeting NF-κB and/or NLRP3 inflammasome.

## 11. Natural Phenolic Compounds in Sepsis and Lung Injury

Sepsis manifestations including cytokine storm, endothelial cell dysfunction, intravascular coagulation, pulmonary, cardiovascular, and renal complications have all been reported in COVID-19 patients [164,165]. Dysregulated immune response and cytokine storm [166], with elevated levels of IL-6, IL-10, and TNF-α, and lymphopenia, correlated with worse outcomes in COVID-19 patients [167]. Therefore, anti-inflammatory and anti-coagulant drugs could be considered to reduce hyperinflammation and incidence of thrombosis, multiple organ failure and death [168].

Experimental models to study sepsis include, for example, cecum ligation and puncture (CLP) and LPS lethal dose, since LPS binds to TLR4 which activates NF-κB and IRF3 pathways inducing the production of proinflammatory cytokines and cellular activation [169,170].

TLR4 is activated by Ebola virus (EBO), vesicular stomatitis virus (VSV), DENV, and SARS-CoV-2 [171,172]. It has been shown that SARS-CoV-2′s spike protein activates TLR4 and triggers IL-1β and IL-6 production by THP-1 cells [172]. In addition, lung tissue injury, caused by SARS-CoV-2 infection, could induce danger associated molecular patterns (DAMPs) which activate TLR-4 and potentiate inflammation [171]. Therefore, the anti-inflammatory activities of natural phenolic compounds could be useful in severe inflammation and lung injury associated with COVID-19.

EGCG is among the natural phenolic compounds that inhibit TLR signaling. EGCG inhibited TLR4 signaling and ameliorated acute lung injury in mice infected with H9N2 influenza virus [173]. Resveratrol was also found to impairs TLR4 and TLR3 pathways independent of MyD88 signaling [174]. In addition, resveratrol suppressed RSV replication, IL-6 secretion and TRIF-TBK1 pathway in 9HTEo cells; human epithelium tracheal cells, that are infected and treated with 100 μM of resveratrol [175]. In vivo, resveratrol (30 mg/kg) reduced RSV titer in the lungs of infected mice, and impaired TLR3-TRIF signaling pathway, alleviating airway hyperresponsiveness and inflammation [176].

Amentoflavone and apigenin were shown to reduce inflammation in sepsis models. In CLP-induced sepsis, amentoflavone treatment (50 mg/kg) protected rats from acute lung injury by decreasing TNF-α and IL-1β levels, impairing NF-κB activity and reducing oxidative stress in the lung tissue [177]. Mice treated with 50 mg/kg of apigenin, 3h before receiving a lethal dose of LPS, showed enhanced survival with decreased lung cell death, and reduced TNF-α production and neutrophil infiltration into the lung tissue. In addition, cardiac function and heart mitochondrial complex I activity were restored in these mice [178].

Acute lung injury (ALI) and acute respiratory distress syndrome (ARDS) are also described in COVID-19 patients, and correlated with worse outcome and higher mortality [179]. Hesperetin demonstrated the ability to suppress inflammatory cytokines production, inflammatory cell infiltration into the lung tissue, and reduced myeloperoxidase and LDH activities in different models of ALI [180,181,182].

Besides the importance of neutrophils in early responses to infections, they can damage tissues and are also involved in sepsis-induced tissue injury [183]. It has been shown that neutrophils accumulate in lungs of severe COVID-19 patients [184]. Neutrophil-extracellular traps (NETs) were detected in high levels in the plasma and lung tissues of COVID-19 patients [185], indicating that neutrophils activation is detrimental in COVID-19 patients. A study has shown that luteolin (30 μM) inhibited oxidative stress, and reduced NETs formation in human neutrophils that were activated with PMA [186]. Amentoflavone impaired oxidative burst in human neutrophils stimulated with PMA and protected human erythrocytes from oxidative hemolysis. These effects were explained by the ability of amentoflavone to inhibit NADPH oxidase and ROS production in human neutrophils and to prevent membrane damage and lipid peroxidation in human erythrocytes [187]. However, more studies are needed to further understand the mechanism by which amentoflavone inhibit neutrophil oxidative burst and erythrocyte lysis.

## 12. Natural Phenolic Compounds in Extrapulmonary Complications of COVID-19

Neurologic symptoms have been described in COVID-19 patients, including anosmia, ageusia, encephalopathy, seizures, encephalitis, stroke, and cognitive disturbance [188,189]. SARS-CoV-2 have been shown to infect neurons and damage the central nervous system (CNS) [190,191]. The detection of low or no viral copies in the brain tissue has been described in a number of COVID-19 cases with neurologic complications [192], and it remains unclear whether the CNS complications are caused by direct infection or inflammation. Seizures are among the neurologic complications that have been reported during and after recovery from SARS-CoV-2 infection [193,194,195]. Brain inflammation, genetic factors, developmental dysfunction, environmental risk and neurological insults are involved in epileptogenesis and seizures susceptibility [196]. Amentoflavone has been described as neuroprotective in experimental models of epilepsy. Amentoflavone suppressed NF-κB activation, decreased production of NO, PGE_2_, IL-1β, and IL-6, prevented hippocampus neurons apoptosis, and decreased epileptic seizures in pilocarpine-treated mice [197]. Moreover, amentoflavone blocked apoptosis, impaired NLRP3 inflammasome activation, and decreased production of IL-18, IL-1β, and TNF-α in brains of pentylenetetrazole-induced kindling mice [151].

The anti-inflammatory, anti-oxidant and anti-apoptotic effects of hesperetin have been related to its ability to protect neuronal [198,199], cardiac [200] and renal tissues [201] in different injury models. Hesperetin ameliorated neuroinflammation, memory, and impaired neuronal apoptosis in vivo [198]. Hesperetin interfered with the TLR4-NF-κB signaling pathway. Accordingly, mice treated with LPS and hesperetin (50 mg/kg) showed decreased brain levels of p-NF-κB, IL-1β and TNF-α compared to mice that received only LPS. The anti-inflammatory and cytoprotective effects of hesperetin were also confirmed in vitro using BV-2, and HT-22 mouse hippocampal neuronal cell line [198].

Acute myocarditis is one of the extrapulmonary complications in COVID-19 patients [202], and is associated with inflammatory cell infiltration into the heart tissue [203]. It has been shown that apigenin prevented myocarditis in an experimental model of autoimmune myocarditis. Treatment with 200 mg/Kg (gavage) of apigenin reduced inflammatory cell infiltration into the heart, decreased TNF-α, IL-2 and IFN-γ, and ameliorated cardiac dysfunction compared to untreated mice [204]. Anti-apoptotic effects of 25 μM of hesperetin was also demonstrated in in vitro LPS-treated H9C2 cardiomyocytes [205], and in a myocardial infarction (MI) model in vivo. Indeed MI mice, treated with 30 mg/kg/day of hesperetin for 8 weeks, showed impaired NF-κB activation, reduced cardiac fibrosis and inflammation compared to untreated MI-mice [200].

**Figure 3:**
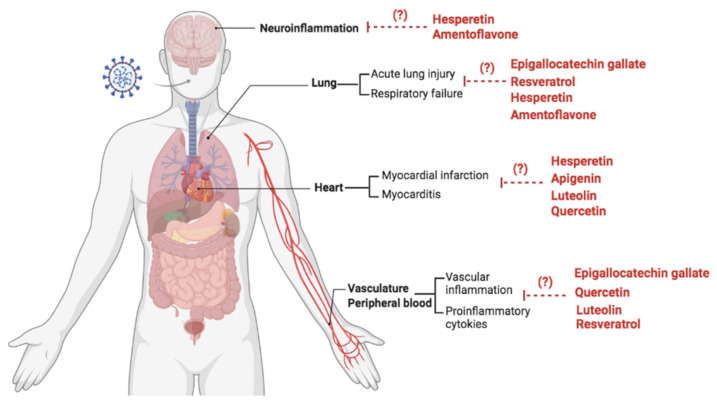
Immunomodulatory actions of natural phenolic compounds for further investigation in SARS-CoV-2 infection (Dashed lines = Inhibition).

Kawasaki-like disease (KD) was also described in COVID-19 pediatric patients [206]. Proinflammatory cytokines are related to hyperinflammation, vasculitis and coronary artery damage in KD patients. Increased TNF-α and IL-1β levels in KD patients result in endothelial cell activation and expression of adhesion molecules which leads to leukocyte adherence and endothelial injury, promoting vasculitis and coronary artery aneurysms [207]. Human coronary arterial endothelial cells, activated with 10 ng/mL of TNF-α, showed enhanced VCAM-1 and ICAM-1 expression, oxidative stress and proinflammatory cytokines production. However, in the presence of 10 μM of resveratrol, expression of ICAM-1, iNOS, and IL-1β were reduced which indicate that resveratrol has anti-inflammatory actions on coronary arterial cells and could be promising in treatment of KD patients [208]. Moreover, quercetin treatment (50 mg/kg) prevented cardiac injury, inflammation and oxidative stress in the heart of streptozocin (STZ) and nicotinamide-induced diabetic rats [209]. Luteolin (10 µM) protected H9C2 cardiomyocytes from inflammation and oxidative stress induced by high glucose concentration. Additionally, reduced inflammation was observed in the heart of STZ-diabetic mice that were treated with 20 mg/kg of luteolin for 15 weeks [210]. Figure 3 illustrates the main mechanisms of the immunomodulatory actions of phenolic compounds discussed in this study.

Most of the compounds discussed in this review are found in foods and beverages of natural origin, such as resveratrol, which is commonly present in wine. However, it is not possible to conclude that a diet based on these foods will result in prevention or improvement of the clinical conditions of affected people by COVID-19. Discussion of this possibility requires carrying out comprehensive studies in populations that have an appropriate diet. For example, the high consumption of wine in France may contribute to the low frequency of coronary heart disease, possibly due to the presence of resveratrol in this drink. This evidence represents the French paradox [211]. However, countries with high consumption of wine, such as France and Italy, had a high number of deaths caused by COVID-19 [212,213]. Therefore, studies using standardized methods with these phytoconstituents are needed to advance the knowledge of their therapeutic potential against COVID-19.

## 13. Conclusions

Among natural phenolic compounds discussed, we highlighted the antiviral effects of quercetin, luteolin, resveratrol, and amentoflavone against coronaviruses as well as their ability to modulate immune response and inflammatory status in a variety of in vitro and in vivo models. Despite the structural complexity of some bioactive compounds, there are perspectives for the development of synthetic analogues with an anticoronavirus and immunomodulator profile, but structurally simpler and easier to obtain using the phytoconstituents in this review as prototypes. In addition, it is possible to manufacture plant products containing a significant amount of these phenolic compounds and use them as potentially therapeutic agents against COVID-19. So, further experimental studies focusing on anti-SARS-CoV-2 and immunomodulatory activities of these compounds are needed.

## Figures and Tables

**Table 1:** Anticoronavirus actions of natural phenolic compounds.

Compound	Mechanism of Action	IC_50_	CC_50_	SI	Experimental Model	Reference
Quercetin	-	198.5 μM	-	-	HCoV-OC43 and NCDCV infection of embryonic bovine lung fibroblasts	[20]
Apigenin, Luteolin, and Catechin	Blockade of early steps of viral life cycle	Apigenin: 0.37–0.74 μM Luteolin: 0.7–1.4 μM Catechin: 37.9–41.3 μM	Apigenin: >185 μM Luteolin: 23.4 μM Catechin: >341.7 μM	Apigenin: 250–500 Luteolin: 16.75–33.5 Catechin: 8.3–9	PEDV infection of Vero cells and Sulforhodamine B assay for cytotoxicity	[21]
Luteolin and Quercetin	Inhibiton of viral entry by binding to S2 domain of S protein and inhibiting fusion	Luteolin: 10.6 μM Quercetin: 83.4 μM	Luteolin: 155 μM Quercetin: 3320 μM	Luteolin: 14.62 Quercetin: 39.8	SARS-CoV live virus and HIV-luc/SARS pseudotyped viral infection of Vero E6 cells	[22]
Hesperetin	Inhibition of SARS-CoV 3CL^pro^	8.3 μM and 60 μM in cell-based and cell-free assays respectively	2718 μM	327.5 and 45.3 in cell-based and cell-free assays respectively	Cell-free assay using recombinant 3CL^pro^ fusion protein and substrate Cell-based assay using recombinant 3CL^pro^–substrate–luciferase fusion protein	[25]
Amentoflavone	Inhibition of SARS-CoV 3CL^pro^	8.3 μM	-	-	FRET assay using commercial 3CL^pro^	[26]
GCG and EGCG	Inhibition of SARS-CoV 3CL^pro^	GCG: 47 μM EGCG: 73 μM	-	-	FRET assay using recombinant 3CL^pro^	[27]
Herbacetin, Rhoifolin and Pectolinarin	Inhibiton of SARS-CoV 3CL^pro^	Herbacetin: 33.17 μM Rhoifolin: 27.45 μM Pectolinarin: 37.78 μM	-	-	FRET assay using recombinant SARS-CoV 3CL^pro^	[28]
Psoralidin and Isobavachalcone	Inhibition of SARS-CoV PL^pro^	Psoralidin: 4.2 ± 1.0 μM Isobavachalcone: 7.3 ± 0.8 μM	-	-	Fluorescence-based assay using a fluorogenic substrate and recombinant SARS-CoV PL^pro^	[29]
Herbacetin, Isobavachalcone, and Helichrysetin	Inhibition of MERS-CoV 3CL^pro^	Herbacetin: 40.59 μM Isobavachalcone: 35.85 μM Helichrysetin: 67.04 μM	-	-	FRET assay using recombinant MERS-CoV 3CL^pro^	[30]
Quercetin	Inhibition of helicase activity of SARS-CoV NSP13	Quercetin: 8.1 μM	-	-	FRET-based assay for the DNA unwinding activity of helicase	[32]
Myricetin and Scutellarein	Inhibition of ATPase activity of SARS-CoV NSP13	Myricetin: 2.71 ± 0.19 μM Scutellarein: 0.86 ± 0.48 μM	-	-	Colorimetric-based ATP hydrolysis assay	[33]
Quercetin	Inhibition of SARS-CoV-2 3CL^pro^	-	-	-	FRET assay using recombinant SARS-CoV-2 3CL^pro^	[34]
EGCG	Inhibition of SARS-CoV-2 3CL^pro^	0.874 ± 0.005 μM	-	-	FRET assay using recombinant SARS-CoV-2 3CL^pro^	[35]
EGCG	Inhibition of viral entry by blocking the binding of SARS-CoV-2 S protein to ACE2	3.77 μM	-	-	Plaque reduction assay using live SARS-CoV-2	[36]
EGCG	Inhibition of endoribonuclease activity of SARS-CoV-2 Nsp15	1.62 µM and 0.2 µM in enzymatic assay and live virus infection assay respectively	-	-	FRET assay using recombinant NSP15 and live SARS-CoV-2 palque reduction assay	[37]
GCG	Inhibition of binding of SARS-CoV-2 N protein to viral RNA	44.4 µM	155.4 µM	3.5	SARS-CoV-2 infection of A549-hACE2 cells	[38]
Herbacetin, Pectolinarin	Inhibition of SARS-CoV-2 3CL^pro^	Herbacetin: 53.90 µM Pectolinarin: 51.64 µM	-	-	FRET assay using recombinant SARS-CoV-2 3CL^pro^	[39]
Myricetin	Inhibition of SARS-CoV-2 3CL^pro^	3.684 ± 0.076 μM	-	-	FRET assay using recombinant SARS-CoV-2 3CL^pro^	[40]
Quercetin	Inhibition of ACE2	4.48 µM	-	-	FRET assay using recombinant ACE2 and Mca-APK(Dnp) as the substrate	[41]
Resveratrol	Inhibition of MERS-CoV viral RNA replication, nucelocapsid protein expression, and MERS-CoV-mediated cell apoptosis Inhibiton of SARS-CoV-2 viral entry	4.48 µM	>200 µM	>45	Live MERS-CoV infection assays such as plaque assay, MTT, and neutral red uptake assay as well as immunofluorescent assay Live SARS-CoV-2 infection of Vero cells	[43] [44]

**Table 3:** Anti-inflammatory effects of natural phenolic compounds in LPS-stimulated monocytes/macrophages.

Compound	Concentration	In Vivo Models Using LPS-Stimulated Monocytes/Macrophages	Effect	Reference
Amentoflavone	60 μM	RAW 264.7 pretreated with amentoflavone 10 min prior to LPS stimulation	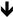 NO 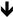 p65 nuclear translocation 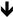 I-κBα degradation	[113]
Herbacetin	50 μM	RAW 264.7 pretreated with herbacetin 30 min prior to LPS stimulation	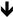 NO 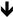 NF-κB activation 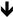 IL-1β and TNF-α levels	[115]
Rhoifolin	100 μM	LPS-stimulated RAW 264.7 in the presence of rhoifolin treatment	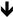 IκBα and IKKβ phosphorylation 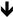 TNF-α, IL-1β, IL-6 and CCL2 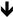 iNOS	[116]
Luteolin	5 μM	LPS-stimulated RAW 264.7 in the presence of luteolin treatment	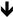 iNOS, NO 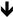 NF-κB nuclear translocation 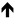 HO-1	[117]
Myricetin	100 μM	RAW 264.7 pretreated with myricetin 1h prior to LPS stimulation	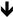 iNOS and COX-2 expression 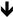 NF-κB p65 nuclear translocation 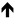 HO-1, Nrf2	[118]
Psoralidin	30 μM	LPS-stimulated RAW 264.7 in the presence of psoralidin treatment	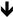 iNOS expression 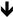 NF-κB nuclear translocation	[119]
Scutellarin	100 μM	RAW 264.7 pretreated with scutellarin 1h prior to LPS stimulation	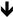 PGE_2_ 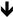 NO 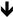 IL-6 and TNF-α expression	[120]
Hesperetin	40 μM	LPS-stimulated RAW 264.7 in the presence of hesperetin treatment	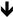 IL-6, IL-1β, TNF-α expression 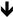 iNOS and COX-2 expression 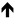 HO-1 and Nrf2	[121]
Apigenin	10 μM 25 μM	LPS-stimulated human monocytes in the presence of apigenin treatment Human THP-1-derived macrophage pretreated with apigenin 2h prior to LPS stimulation	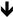 IL-8, IL-1β, TNF-α 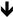 p65 phosphorylation 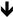 ERK1/2 phosphorylation 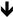 NF-κB activation 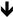 IL-6 and IL-1β expression	[122] [123]
Catechin	40 μM	Human THP-1-derived macrophage pretreated with catechin 4h prior to *Porphyromonas gingivalis* infection	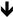 NF-κB activation 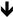 TNF-α and IL-1β production	[124]
EGCG GCG	0.3–30 μM	Murine peritoneal macrophages and J774.1 macrophages pretreated with EGCG or GCG 24h prior to LPS stimulation	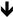 IκBα degradation 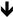 NF-κB activation 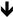 IL-12p40 and TNF-α production	[127]
Quercetin	20 μM	RAW 264.7 pretreated with quercetin 30min prior to LPS stimulation	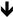 I-κB phosphorylation 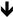 NF-κB nuclear translocation 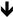 NO, PGE_2_, TNF-α, IL-6, IL-1β and GM-CSF production 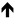 HO-1	[134]
Quercetin Catechin	3 μM 75 μM	LPS-stimulated RAW 264.7 in the presence of quercetin and catechin treatment	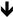 NF-κB p65 phosphorylation 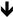 iNOS, COX-2 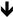 TNF-α and IL-1β secretion	[135]
Resveratrol	100 μM	Human PBMC pretreated with resveratrol 2h prior to LPS stimulation	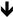 TNF-α, IL-6, IFN-γ, G-CSF, GM-CSF production 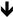 CCL-2, CCL-5, CXCL5 production	[137]
Isobavachalcone	5 μM	LPS-stimulated BV-2 in the presence of isobavachalcone	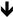 NF-κB activation 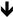 TNF-α, IL-6, IL-1β and iNOS expression	[139]
Scutellarin	139.7 μM	LPS-stimulated BV-2 in the presence of scutellarin	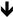 NF-κB-p65, p38, JNK, and AKT phosphorylation 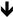 TNF-α, IL-1β, IL-6 and NO production	[140]

The compounds in the table are in the order in which the compounds are presented in the section *Effects of Natural Phenolic Compounds on NF-κB Pathway and Inflammation*. 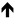 Increased or 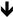 decreased.

## Data Availability

Not applicable.

## Abbreviations

229E	Human coronavirus-229E
(+ss)	Positive single-stranded
3CL^pro^	3-Chymotrypsin-like protease
ACE2	Angiotensin converting enzyme 2
ALI	Acute lung injury
APCs	Antigen presenting cells
ARDS	Acute respiratory distress syndrome
ASC	Caspase-recruitment domain
BMDMs	Bone-marrow derived macrophages
CCL5	Chemokine (C-C motif) ligand 5
CLP	Cecum ligation and puncture
CNS	Central nervous system
CoVs	Coronaviruses
COVID-19	Coronavirus disease 2019
CRP	C-reactive protein
CTLs	Cytotoxic T lymphocytes
DAMPs	Danger associated molecular patterns
DENV	Dengue virus
EBO	Ebola virus
EGCG	Epigallocatechin gallate
ER	Endoplasmic reticulum
GCG	Gallocatechin gallate
HCoVs	Human coronaviruses
HCoV-OC43	Human coronavirus-OC43
HCV	Hepatitis C virus
KD	Kawasaki-like disease
HKU1	Human coronavirus-HKU1
Hmpv	Human metapneumovirus
HO-1	Heme oxygenase-1
ICAM-1	Intercellular adhesion molecule-1
IFN	Type I interferon
iNOS	Inducible nitric oxide synthase
IRF	Interferon-regulatory factor 3/7
ISGs	IFN-stimulated genes
JNK	Jun N-terminal kinase
LDH	Lactate dehydrogenase
LPS	Lipopolysaccharide
MARCO	Macrophage receptor with collagenous structure
MCP-1	Chemokine monocyte chemotactic protein-1
MERS-CoV	Middle east respiratory syndrome-coronavirus
MyD88	Myeloid differentiation primary response 88
NCDCV	Neonatal calf diarrhea coronavirus
NETs	Neutrophil-extracellular traps
NF-κB	Nuclear factor kappa B
NK	Natural killer
NL63	Human coronavirus-NL63
NLR	NOD-like receptor
NLRP3	Pyrin domain-containing 3
NSP13	SARS-CoV nonstructural protein 13
NTHi	Nontypeable *Haemophilus influenza*
OxLDL	Oxidized low-density lipoprotein
PBMCs	Peripheral blood mononuclear cells
PEDV	Porcine epidemic diarrhoea coronavirus
PL^pro^	Papain-like protease
PMA	Phorbol myristate acetate
Poly I:C	Polyinosinic–polycytidylic acid
PRR	Patter recognition receptors
RIG-I	Retinoic acid-inducible gene I
ROS	Reactive oxygen species
RSV	Respiratory syncytial virus
SARS-CoV	Severe acute respiratory syndrome-coronavirus
SARS-CoV-2	Severe acute respiratory syndrome-coronavirus 2
VCAM-1	Vascular cell adhesion protein-1
VSV	Vesicular stomatitis virus
TLR	Toll-like receptors
TRIF	TIR-domain-containing adapter-inducing interferon-β (TRIF)
TXNIP	Thioredoxin-interacting protein
IC_50_	Concentration that results in 50% inhibition
CC_50_	Concentration that reduces cell viability by 50%
